# From Native Glycosaminoglycans to Mimetics: Design, Mechanisms, and Biomedical Applications

**DOI:** 10.3390/biom15111518

**Published:** 2025-10-27

**Authors:** Fabian Junker, Sandra Rother

**Affiliations:** Institute of Biophysics, Center of Integrative Physiology and Molecular Medicine (CIPMM), Saarland University, 66421 Homburg, Germany

**Keywords:** glycosaminoglycan mimetics, glycosaminoglycans, heparan sulfate mimetics, glycan/protein interaction, heparinase inhibitors, regenerative medicine, cancer therapy, anti-inflammatory agents, antiviral agents

## Abstract

Glycosaminoglycans (GAGs) are essential regulators of numerous biological processes through their interactions with growth factors, chemokines, cytokines, and enzymes. Their structural diversity and heterogeneity, however, limit reproducibility and translational use, as native GAGs are typically obtained from animal-derived sources with notable batch-to-batch variability. To overcome these challenges, a wide range of GAG mimetics has been developed with the aim of replicating or modulating the biological functions of native GAGs while offering improved structural definition, accessibility, and therapeutic potential. Polysaccharide-based GAG mimetics, including derivatives of heparan sulfate, hyaluronan, dextran, and other natural glycans, represent one major strategy, whereas non-saccharide-based mimetics provide alternative scaffolds with enhanced stability and selectivity. Both approaches have yielded compounds that serve as valuable tools for dissecting GAG/protein interactions and as candidates for therapeutic development. Biomedical applications of GAG mimetics span diverse areas such as cancer, cardiovascular and inflammatory diseases, bone and cartilage regeneration, wound healing, and infectious diseases. This mini-review summarizes key developments in the design and synthesis of GAG mimetics, highlights their potential biomedical applications, and discusses current challenges and future perspectives in advancing them toward clinical translation.

## 1. Introduction

This review provides an overview of the current state of glycosaminoglycan (GAG) mimetics, highlighting their structural design, mechanisms of action, and biomedical applications. Native GAGs are linear polysaccharides with essential regulatory functions in processes such as cell proliferation, angiogenesis, inflammation, and tissue repair, mediated through interactions with a broad range of proteins, including growth factors, chemokines, and enzymes [1,2,3,4]. Recent advances in glycan microarray technologies have enabled systematic mapping of GAG/protein interactions, thereby expanding our understanding of sulfation-specific binding motifs [5]. However, their inherent heterogeneity, dependence on animal-derived sources, and limited availability pose significant challenges for reproducibility and large-scale biomedical use. To address these limitations, chemically synthesized, chemo-enzymatically produced, and bioengineered GAG mimetics have emerged as promising alternatives. These compounds aim to replicate or modulate the biological functions of native GAGs while offering improved structural definition, accessibility, and therapeutic potential. Importantly, GAG mimetics are increasingly investigated not only as research tools for dissecting GAG/protein interactions but also as candidates for clinical translation in diverse fields ranging from regenerative medicine to cancer therapy [6]. This review first outlines polysaccharide-based mimetics, followed by non-saccharide approaches, and concludes with a discussion of their biomedical applications, clinical translation, and safety considerations.

## 2. Learning from Nature: Synthesis and Interaction of Native GAGs

**Figure 1:**
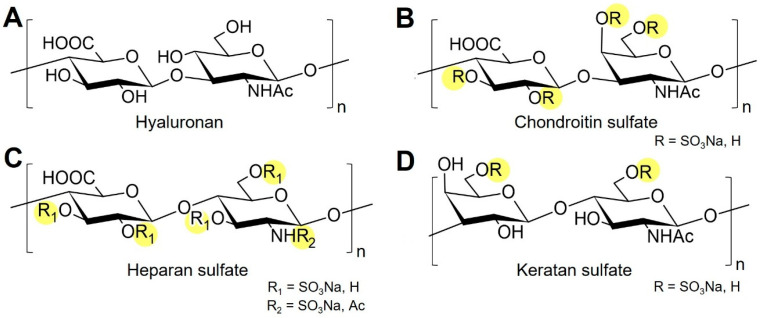
Representative disaccharide structures of major GAGs. (**A**) HA consists of β-D-glucuronic acid (GlcA) and β-D-N-acetylglucosamine (GlcNAc) linked by alternating β(1→3) and β(1→4) bonds. (**B**) CS contains β-D-glucuronic acid (GlcA) and β-D-N-acetylgalactosamine (GalNAc) units joined by β(1→3)/β(1→4) linkages, variably sulfated at C4 and/or C6 of GalNAc. (**C**) HS consists of alternating α-L-iduronic or β-D-glucuronic acid (IdoA/GlcA) and α-D-glucosamine (GlcN) residues connected through α(1→4)/β(1→4) linkages and extensively N- and O-sulfated. (**D**) Keratan sulfate is composed of β-D-galactose (Gal) and β-D-N-acetylglucosamine (GlcNAc) linked by β(1→3)/β(1→4) bonds and O-sulfated mainly at C6. Sulfated or potentially sulfated groups are highlighted in yellow. Structural differences in monosaccharide composition and sulfation pattern define the diversity of GAG function.

GAGs are a family of linear polysaccharides composed of repeating disaccharide units, typically of an amino sugar (glucosamine or galactosamine) and a uronic acid (glucuronic (GlcA) or iduronic acid (IdoA)) or galactose in the case of keratan sulfate (KS); the major mammalian types include heparan sulfate (HS), heparin, chondroitin sulfate (CS), dermatan sulfate (DS), KS, and the non-sulfated hyaluronan (HA) (Figure 1). They are found ubiquitously on cell surfaces (as components of proteoglycans), in the extracellular matrix (ECM), and in various bodily fluids, where their distribution is highly tissue- and cell-type specific. The biosynthesis of sulfated GAGs (except for HA) takes place in the Golgi apparatus via sequential action of glycosyltransferases, epimerases, and sulfotransferases, starting from core protein linkage and elongation of disaccharide repeats, followed by modifications such as acetylation, sulfation (at multiple positions), and epimerization (e.g., conversion of GlcA to IdoA in HS/DS) [7,8]. These multiple enzymatic steps, which are under genetic, developmental, and environmental regulation, cause each GAG chain to differ in length, sulfation pattern, acetylation state, and epimerization. This intrinsic “microheterogeneity” (and sometimes macro variation between tissues or species) is a major reason for the challenges in consistency, purification, and functional characterization of native GAGs [9]. At the molecular level, GAG interactions with proteins are dominated by ionic interactions between negatively charged sulfate/carboxylate groups on the GAGs and clusters of positively charged amino acid residues (lysine, arginine) on proteins, hydrogen bonding, and van der Waals contacts. Furthermore, the three-dimensional conformation of the GAG (including flexibility/rigidity, orientation of sulfates, conformation of uronic acid residues) also contributes importantly to affinity and specificity [10]. These interactions enable GAGs to modulate a wide array of biological processes: growth factor activity, cell adhesion, receptor binding, morphogen gradients, ECM assembly, and even protection of ligands from proteolytic degradation [11].

## 3. Rationale for GAG Mimetics and Synthetic and Bioengineering Strategies

The extensive structural diversity of native GAGs as well as their limited availability due to the requirement of extraction from different animal-derived tissues are drawbacks limiting their broad use in biomedical applications. The variability regarding their chain length and especially their degree of modification due to acetylation, sulfation, and epimerization reactions give rise to a broad heterogeneity of native GAGs. As a result, notable batch-to-batch variations need to be considered while working with native GAGs [12]. To overcome these limitations, several GAG mimetics have been developed to analyze their interaction for HS-binding proteins, to modify the binding and release profiles of surfaces and biomaterials, and as therapeutics to interfere with disorders accompanied by altered HS/protein interactions. In this review, we define a GAG mimetic as a non-native, chemically defined molecule or engineered polymer (saccharide or non-saccharide) that reproduces or modulates mammalian GAG/protein interactions. This category includes (i) chemically modified natural GAG backbones, such as sulfated hyaluronan (sHA), which does not occur naturally but imitates the functions of sulfated GAGs; (ii) fully synthetic oligosaccharides, exemplified by fondaparinux, which reproduces the antithrombin-binding pentasaccharide of heparin; and (iii) non-saccharide scaffolds, such as sulfated cyclitols or flavonoids, designed to mimic sulfate spacing and charge density of sulfated GAGs. By contrast, native mammalian GAGs (e.g., HS, CS, DS, KS, HA) and bioengineered polysaccharides identical to them (e.g., recombinant CS-A or CS-C produced in *E. coli*) are not considered mimetics here but rather native equivalents. Natural GAG analogs from non-mammalian sources (e.g., fucosylated CS from marine organisms) are discussed when used experimentally as surrogates for mammalian GAGs.

Different strategies, such as the chemical and chemo-enzymatic synthesis [13,14] and the bioengineered production [15] of GAG mimetics, are currently explored. The use of GAG mimetics with defined sulfation motifs should improve the understanding and dissecting of structural binding motifs required for the pleiotropic functions of native GAGs. Novel synthetic strategies now also explore conjugation approaches, such as cholesterol-modified GAG mimetics, to improve oral bioavailability and delivery [16]. Ideally, GAG mimetics as druggable compounds should be (i) homogeneous with (ii) enhanced selectivity for HS interaction partners compared to native HS, (iii) easy to synthetize, (iv) cost-effective in production, and (v) available in large quantities, with (vi) no or only few off-target and side effects. Overall, GAG mimetics can be grouped into polysaccharide-based derivatives and non-saccharide-based compounds.

### 3.1. HS Mimetics Based on Modified Polysaccharides

Different polysaccharides have been explored for chemical modification to derive HS mimetics. The oligosaccharide mixture phosphomannopentaose sulfate (PI-88) is the sulfated reaction product of yeast-derived phosphomannan containing 60% tetra- and 30% pentasaccharides [17]. Besides its function as a heparinase inhibitor and its anticoagulative potential, it has been reported to bind HS-binding proteins such as vascular endothelial growth factor-A (VEGF-A), and fibroblast growth factor-1 (FGF-1) and -2 (FGF-2), thereby competing with native HS for these bioactive mediator proteins [17,18]. Further PI-88-related HS mimetics have been developed with improved pharmacokinetic properties and decreased anticoagulative activity [19,20]. A rationally designed octasaccharide synthesized by Kuhnast et al. was shown to inhibit the binding of vascular VEGF-A, FGF-2, platelet derived growth factor-β (PDGF-β), and stromal cell-derived factor-1α (SDF-1α) to heparin, with IC50 values in the low nM range [21].

Moreover, different bacteria-derived polysaccharides have been used as starting materials for the synthesis of GAG mimetics. For example, Matou et al. over-sulfated an exopolysaccharide secreted by Alteromonas infernus. Compared to the non-sulfated polysaccharide, this GAG mimetic stimulated the proliferation, migration, and tube formation of human endothelial cells in vitro, suggesting an interplay with angiogenic factors such as FGF-2 and VEGF-A [22]. A biotechnological–chemical process developed by the Schiraldi group gave rise to over-sulfated CS-like polysaccharides [23]. Thereby, a chondroitin-like polysaccharide was extracted after synthesis by *E. coli* O5:K4:H4 and sulfated using the SO3-pyridine complex [24]. ELISA studies showed a higher VEGF-A-binding capacity for surfaces with immobilized over-sulfated CS derivatives compared to native GAGs [25].

Beyond chemo-enzymatic routes, recent synthetic biology advances achieved the complete in vivo biosynthesis of sulfated chondroitin in *E. coli*. This was achieved by co-expressing the chondroitin backbone pathway together with the PAPS sulfate donor system and functional sulfotransferases, yielding defined CS motifs directly in cells [26]. We therefore distinguish bioengineered natural CS (same as mammalian CS) from chemically modified CS-like polymers (e.g., over-sulfated derivatives), which we classify as GAG mimetics in this review [27]. Pioneering CS glycomimetics from the de Paz/Nieto group introduced aromatic substituents and multivalent presentations to enhance affinity for CS-binding proteins such as midkine/pleiotrophin, establishing design principles later adopted broadly in GAG mimetic libraries [28,29].

Sheng et al. described the synthesis of HS glycomimetics based on a core disaccharide precursor, which is later used to generate differently sulfated HS mimetics. In particular, a trisulfated glycopolymer strongly competed with heparin for binding to CC-chemokine ligand 5 (CCL5), a proinflammatory chemokine [30].

**Figure 2:**
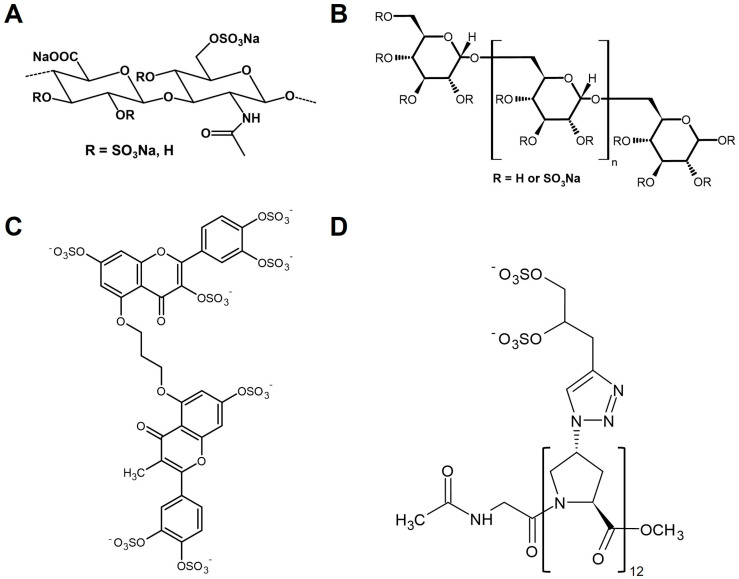
Structures of selected GAG mimetics. (**A**) Sulfated hyaluronan (sHA), a chemically modified hyaluronan; (**B**) DS-500, a highly sulfated dextran; (**C**) G2.2, a synthetic small-molecule mimetic; and (**D**) {Z}_12_, a glycomimetic with defined sulfation patterns.

A further widely explored group of GAG mimetics is based on HA, which can be produced on a large scale by Streptococcus zooepidemicus. Several reviews summarize the various strategies for HA modification and functionalization [31,32]. It is of note that especially highly sulfated hyaluronan (sHA, Figure 2A) derivatives showed even higher binding levels for various biologically active proteins, such as growth factors like transforming growth factor-β1 (TGF-β1) [33], bone morphogenic protein-2 (BMP-2) [34] and -4 (BMP-4) [35], VEGF-A [36], heparin-binding epidermal growth factor-like growth factor (HB-EGF) [37], inhibitors of matrix degradation and angiogenesis-like tissue inhibitor of metalloproteinases-3 (TIMP-3) [38], and modulators of receptor activator of NF-κB ligand (RANKL)/RANK and Wnt signaling such as osteoprotegerin [39] and sclerostin [40], in surface plasmon resonance (SPR) studies compared to native GAGs such as CS and heparin or carboxymethylated HA derivatives. Furthermore, sHA was shown to inhibit the activity of mouse and human heparinase to enzymatically degrade fluorescein-labeled HS [41]. Modeling studies pointed out an electrostatic interaction between an sHA tetrasaccharide and the heparin-binding domain-1 of heparinase. Computational and structural studies of the HS mimetic pixatimod (PG545) further illustrate how defined sulfation motifs can modulate viral spike protein interactions, underscoring the translational potential of engineered GAG mimetics [42].

Dextran, a branched polysaccharide, is frequently chemically functionalized with sulfate, carboxylate, or acetate groups to generate GAG mimetics, which are commonly employed to investigate interactions with heparin-binding proteins such as stromal cell-derived factor-1α (SDF-1α) [43]. While carboxymethyl–dextran sulfate GAG mimetics had no influence on the expression of HS biosynthetic enzymes, they were shown to reduce the mRNA and protein level of heparinase-1 in Huh7 human hepatoma cells. However, there were no detectable differences in the heparinase activity in the cell lysates after treatment with these dextran derivatives using a heparin/FGF-2 detection system. In addition, GAG mimetics like dextran sulfate-500 (DS-500, Figure 2B), suramin, and pentosan polysulfate are used to evaluate the role of sulfated GAGs as cofactors of prion protein accumulation, highlighting the role of sulfate residues in altering protein interactions and functions [44]. GAG mimetics from the RGTA^®^ family act as ReGeneraTing Agents and are often dextran-based. Dextran sulfate has also been increasingly explored as a nanocarrier for bioactive molecules, broadening its potential beyond anticoagulation to applications in drug delivery and tissue engineering [45]. Moreover, clinical observations with RGTA^®^ formulations demonstrate resistance against enzymatic degradation and promising effects in wound healing [46].

In addition, other natural glycans, such as alginate, fucans, xylan, chitosan, and levan, have been chemically modified to obtain GAG mimetics [47,48,49,50,51]. Erginer et al. used sulfated derivatives of levan, a natural polymer composed of β(2-6)-linked fructose, as heparin mimetic glycan. They showed concentration-dependent anticoagulative activity via thrombin inhibition, suggesting a potential for cardiac tissue engineering applications [52]. 6-O-sulfated chitosan was crosslinked to fabricate wound dressings, which were able to scavenge interleukin-6 (IL-6) and TGF-β1 and promote vascularization and re-epithelialization of full-thickness wounds in vivo [53].

Beyond chemically modified GAG mimetics, GAG-functionalized nanomaterials represent an emerging class of bioactive scaffolds. For instance, chitosan- and HA-modified nanocomposites have demonstrated both antimicrobial properties and enhanced wound closure in murine models [54]. Other studies showed that heparin mimetic sulfated alginate carriers can sequester and sustain the release of heparin-binding growth factors (e.g., VEGF, FGF-2, CTGF/IGF-I), while dextran sulfate–based nanoformulations display intrinsic antibacterial activity [51,55,56,57]. These examples underscore the translational potential of combining native GAG structures with nanotechnology to address challenges in infection control and tissue repair.

### 3.2. Non-Saccharide-Based HS Mimetics

A detailed overview of different sulfated non-saccharide-based GAG mimetics was given by Afosah and Al-Horani [58]. In particular, exploiting the GAG/protein interface by drug discovery programs is a promising option for developing novel drug candidates modulating GAG-dependent protein functions. Synthetic HS mimetics composed of sulfated cyclitol subunits coupled by different linkers have been developed by Freeman et al. as highly stable compounds [59]. SPR interaction studies showed the inhibitory potential of these HS mimetics to interfere with the binding of the growth factors FGF-1, FGF-2, and VEGF-A and the chemokine IL-8 to heparin surfaces. Furthermore, those sulfated cyclitols, which resembled pentasaccharides, were as potent as PI-88 for inhibiting the activity of the HS-degrading enzyme heparinase, while all sulfated HS mimetics blocked the activity of cathepsin G. Current efforts in small-molecule discovery have yielded novel heparinase inhibitors with improved potency and selectivity, reinforcing the promise of non-saccharide scaffolds [60]. Structure–activity studies further confirm that such mimetics can mimic critical sulfate spacing requirements while offering higher stability than native saccharides [16].

Raman et al. reported the development of polysulfonated small-molecule GAG mimetics as angiogenesis inhibitors competing with cell-surface HS for the binding of pro-angiogenic proteins [61]. Here, the authors found that the inhibitory activity of these GAG mimetics towards vascular tube formation required two sulfate residues at a distance of 5–10 Å and did not correlate with the number of sulfate groups. A screening of 53 compounds as potential sulfated non-saccharide GAG mimetics for the selective targeting of colon cancer stem cells identified compound G2.2 (Figure 2C), a dimeric sulfated flavonoid, as the lead drug for inhibiting the spheroid formation of cancer cells, with IC_50_ values in the µM range [62]. Mechanistical studies suggested that G2.2 induced cancer cell apoptosis and reduced their ability for self-renewal. These data were confirmed by in vivo experiments that demonstrated that the inhibitory effect of G2.2 on colon cancer stem cells is mediated through p38 MAP kinase activation [63].

A further strategy for the synthesis of GAG mimetics is based on polyproline residues via amide coupling conjugated sulfate residues. Polyproline-based GAG mimetics were thus obtained that model the length and periodicity of GAG disaccharide units without showing any factor IIa activity [64]. The lead molecule {Z}_12_ (Figure 2D) showed comparable activity to heparin in reducing hematogenous metastasis in mice, which was suggested to be primarily associated with the inhibition of P-selectin.

In addition, surfen (bis-2-methyl-4-amino-quinolyl-6-carbamide), a synthetic bis-quinoline derivative, acts as a small-molecule antagonist of HS/protein interactions and has been widely employed as a research tool to probe HS-dependent signaling pathways [65].

## 4. Selected Biomedical Applications of GAG Mimetics

**Figure 3:**
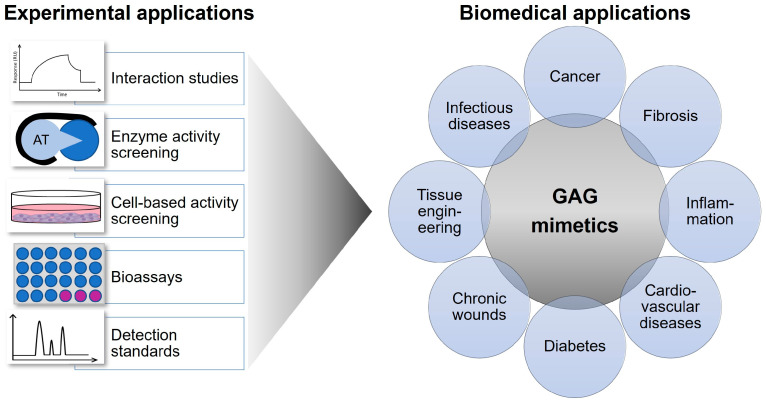
Potential experimental and biomedical applications of GAG mimetics. The schematic highlights both the methodological approaches and biomedical fields in which GAG mimetics are applied. On the left, typical experimental assays are shown: interaction studies (e.g., binding kinetics), enzyme activity screening (e.g., antithrombin/heparinase assays), cell-based activity screening (cell culture models), bioassays (high-throughput activity profiling), and detection standards (analytical characterization). On the right, biomedical application areas are summarized: GAG mimetics are explored, for example, in cancer therapy, fibrosis, inflammation, cardiovascular diseases, diabetes, chronic wound healing, tissue engineering, and infectious diseases.

Due to the broad functions of HS chains in orchestrating key biological processes via their interaction with mediator proteins, various HS mimetics have been explored for biomedical applications [66] (Figure 3). For example, recent work confirmed the antiviral activity of pixatimod (PG545) through direct interference with the SARS-CoV-2 spike protein, highlighting the broader utility of GAG mimetics in infectious diseases [42]. Furthermore, updated reviews on heparinase inhibitors provide compelling evidence for their role in reducing cancer progression and metastasis [52]. A summary of studies focusing on the application of heparin derivatives and HS mimetics in cancer therapy is given in Refs. [67,68], while the effects of sHA derivatives in the field of bone and skin regeneration are reviewed in Refs. [69,70,71,72]. Table 1 highlights some currently studied potential biomedical applications of polysaccharide-based GAG mimetics, and Table 2 summarizes the biomedical uses of selected non-saccharide-based GAG mimetics.

## 5. Glycomimetics in Late-Stage Development and Clinical Translation

**Figure 4:**
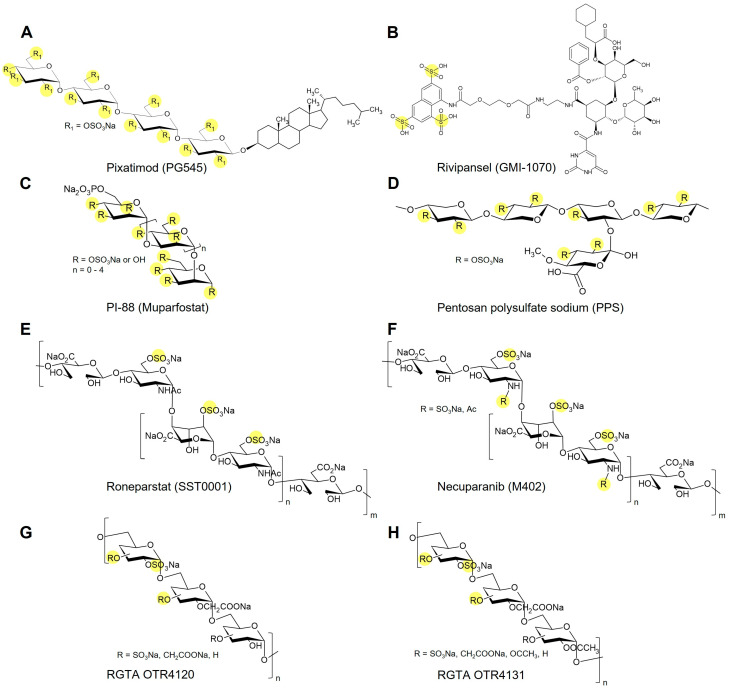
Selected GAG mimetics in late-stage development and clinical translation. Sulfated or potentially sulfated regions are highlighted in yellow. (**A**) Pixatimod (PG545) is a sulfated tetrasaccharide conjugated to a cholestanyl aglycone that combines HS-like interactions with improved lipophilicity; evaluated in oncology and antiviral studies. (**B**) Rivipansel (GMI-1070) is a multivalent glycomimetic bearing carboxylate and sulfate groups that inhibit selectin-mediated adhesion; developed for vaso-occlusive crises in sickle cell disease. (**C**) PI-88 (Muparfostat) is a phosphosulfated oligosaccharide heparanase inhibitor derived from yeast mannan; evaluated in hepatocellular carcinoma treatment. (**D**) Pentosan polysulfate sodium (PPS) is a semisynthetic, highly sulfated heparin analog with anticoagulant and anti-inflammatory activity; approved for interstitial cystitis/bladder pain syndrome. (**E**) Roneparstat (SST0001) is a glycol-split heparin derivative that inhibits heparinase; investigated in clinical trials for the treatment of multiple myeloma. (**F**) Necuparanib (M402) is a HS mimetic that modulates tumor-associated signaling; evaluated in clinical studies for pancreatic cancer therapy. The ReGeneraTing Agents OTR4120 (**G**) and OTR4131 (**H**) are engineered polysaccharides with sulfate and carboxylate substitutions mimicking HS binding to growth factors and ECM components; studied in regenerative medicine and tissue repair.

Several glycomimetics have advanced beyond preclinical evaluation and entered late-stage development or clinical testing (Figure 4). The synthetic pentasaccharide fondaparinux (Arixtra) exemplifies a clinically approved GAG mimetic that reproduces the heparin antithrombin-binding sequence with single-entity chemical definition and predictable pharmacology [95,96]. PI-88 (muparfostat), a sulfated oligosaccharide mixture from yeast phosphomannan, reached Phase III trials in hepatocellular carcinoma as a heparanase inhibitor, though further development was halted due to limited efficacy [18,20,97]. A next-generation derivative, pixatimod (PG545), combines a sulfated saccharide backbone with a lipophilic moiety, improving stability and pharmacokinetics. It has completed Phase I/II oncology trials and showed antiviral activity against SARS-CoV-2 in preclinical models [76,79,98,99,100]. In regenerative medicine, RGTA formulations (e.g., OTR4120, OTR4131), based on dextran sulfate, have shown promising results in early clinical studies on chronic wounds and tissue repair [46,101]. Among non-saccharide mimetics, rivipansel (GMI-1070), an E-selectin antagonist, advanced to Phase III trials for vaso-occlusive crises in sickle cell disease. Although the primary endpoint was not achieved, clinical data confirmed safety and biological activity [91]. The approved pentosan polysulfate sodium (PPS) is a heparin-like polysulfate that is routinely used to treat interstitial cystitis/bladder pain syndrome, where it acts as a replacement for the damaged urothelial GAG layer, as supported by clinical trials [102,103]. Roneparstat (SST0001), a non-anticoagulant, glycol-split heparin, showed heparanase inhibition and clinical activity in a Phase I multiple-myeloma study [104]. Necuparanib (M402), a heparan sulfate mimetic, completed Phase I and Phase II trials in pancreatic cancer. Although the development program was later discontinued, these studies yielded valuable data on pharmacokinetics, dosing, and biomarker correlations [105]. Sevuparin, a low-anticoagulant heparinoid with anti-adhesive activity, advanced to clinical testing in sickle cell vaso-occlusive crises and as adjunct therapy in malaria [106,107]. These agents illustrate both feasibility and remaining challenges for translating structurally defined GAG mimetics to the clinic.

## 6. Safety and Immunogenicity Considerations

The translational success of GAG mimetics critically depends on their safety and immunological profile. The heparin contamination crisis of 2007/2008, which was traced to over-sulfated CS, underscored the risk of adverse immune reactions when structural heterogeneity or impurities are present [108,109]. Fully synthetic, single-entity agents (e.g., fondaparinux) demonstrate that chemical definition and homogeneity can reduce unpredictability relative to complex mixtures [95,96]. Defined mimetics, however, can display favorable or even beneficial immunomodulatory properties. For example, sulfated hyaluronan (sHA) has been shown to reduce pro-inflammatory macrophage functions, attenuate NF-κB signaling, and promote tissue repair in diabetic wound models, suggesting that it can mitigate rather than exacerbate inflammatory responses [110,111,112]. Similarly, pixatimod (PG545) not only functions as a heparinase inhibitor but also exerts immune-regulatory effects, including suppression of effector T-cell activity and promotion of regulatory T-cells [76,113]. Clinical experience with rivipansel (GMI-1070) further indicates good tolerability across multiple trials, with no major immune-related safety signals despite lack of efficacy in Phase III. In regenerative applications, RGTAs like OTR4120 have been reported to reduce inflammation and fibrosis while accelerating wound closure [46,114,115]. Together, these examples illustrate that the immunological impact of GAG mimetics varies with their chemical design. While polydisperse or contaminated preparations carry risk, structurally defined mimetics may offer anti-inflammatory or immune-modulatory benefits in addition to their primary biological activity.

## 7. Conclusions

Recent technological advances for the complex analysis of glycans foster the continuous expansion of our knowledge about GAGs. In particular, computational simulation studies targeting the GAG/protein interface combined with glycan array technologies will help to rationally engineer novel classes of GAG mimetics that can function, for example, as artificial decoy receptors to control dysregulated cell signaling or prevent cell attachment of pathogens, which would advance, e.g., antiviral drug developments. The integration of these in silico strategies with scalable synthesis approaches should ultimately pave the way for clinically viable, structurally defined mimetics. The goal is still to increase the specificity and to allow for a large-scale production of defined GAG mimetics in order to promote their broad biomedical application.

## Figures and Tables

**Table 1:** Studies investigating biomedical applications of saccharide-based GAG mimetics.

Target Protein	GAG Mimetic	Cells/Model	Outcome/Limitation	Ref.
Fibrosis				
TGF-β1	sHA	Human dermal fibroblasts Collagen/sHA-based surface coatings	Reduced myofibroblast differentiation sHA blocked TGF-β1 mediated α-smooth muscle actin, collagen, and fibronectin expression (in vitro)	[73]
-	Sulfated alginate	Sulfated alginate microbeads Human-induced pluripotent stem cell-derived hepatocytes C57BL/6 J mice	Reduced pericapsular fibrotic outgrowth on microbeads with sulfated alginate compared to non-sulfated microbeads (ex vivo)	[74]
Inflammation				
CCL20, L-selectin	2,4-O-di- sulfated iduronic acid (Di-S-IdoA)	Glycan micro array Ovalbumin-induced asthma model in wild-type mice	Di-S-IdoA binds CCL20, inhibits the HS/CCL20 interaction, blocks binding of L-selectin to F2 endothelial cells Reduced total number of leukocytes and decreased airway inflammation in ovalbumin-challenged wild-type mice (in vivo)	[75]
ERK1/2 signaling	PG545	Mouse primary T cells Model of Th1/Th17-dependent inflammation: Methylated bovine serum albumin-induced delayed-type hypersensitivity (DTH) mice	Enhanced induction of anti-inflammatory regulator T-cells Inhibition of ERK1/2 signaling Blocks Th17 polarization in vitro and in vivo	[76]
Cancer				
CCL5	Carboxylated dextran sulfates OTR4120, OTR4131	Human hepatoma cells Huh7	Direct binding of GAG mimetics to CCL5 Inhibited CCL5-induced cell migration and invasion (in vitro)	[77]
Growth factors (not specified)	Sulfated alginate (degree of substitution 22.0, 2.7)	Human lung adenocarcinoma cells (H1792) Mouse lung adenocarcinoma cells (MDA-F471)	Decreased migration of lung adenocarcinoma cells in 2D-scratch assay Inhibited sphere formation Decreased expression of CCL20 Suggested scavenging of growth factors required for proliferation (not specified, analyzed) (in vitro)	[78]
FGF-1, FGF-2, VEGF-A, heparinase, TLR9	PG545	Human umbilical vein endothelial cells (HUVECs) Rat aortic rings in Matrigel Patients with metastatic colorectal/pancreatic cancer, solid tumors	SPR binding affinities for FGF-1, FGF-2, VEGF-A in the nM-range Ki for heparinase: 6.1 ± 2.5 nM Inhibition of HUVEC tube formation Reduced microvessel outgrowths from aortic ring explants Combination with pixatimod showed signs of clinical benefit for patients with colorectal cancer In vitro, ex vivo, phase 1b clinical trial	[79,80]
Diabetes				
Proinflammatory cytokines (IL-1β, TNF-α, IFN-γ)	Low- molecular- weight dextran sulfate	β-cells from dispersed human and mouse islets Prediabetic female NOD mice	Reduced proinflammatory cytokine-induced signaling and β-cell death Increased heparan sulfate proteoglycan staining in islets Daily treatment i.p. reduced insulitis and β-cell death in NOD mice No long-term prevention of diabetes in NOD mice after four weeks of treatment (in vitro, in vivo)	[81]
Cardiovascular diseases				
FGF-2, VEGF-A	Dextran sulfate OTR4131	Human endothelial progenitor cells from umbilical cord blood Female NOD/SCID mice	Enhanced colony formation efficiency, adhesion, and migration of endothelial progenitor cells Enhanced mitogenic effects of FGF-2 and VEGF-A under low serum conditions No effects on tube formation on Matrigel (in vitro and vivo)	[82]
Infectious diseases				
SARS-CoV-2	PG545	SARS-CoV-2 spike protein receptor-binding domain (S1 RBD) Clinical isolates of SARS-CoV-2 Monkey Vero E6 cells Human bronchial epithelial cells A549 K18-hACE2 mouse model	Direct binding of PG545 to S1 RBD Blocked binding of SARS-CoV-2 S1 RBD to A549 cells and binding to ACE2 protein Reduced infection of A549 and Vero E6 cells with SARS-CoV-2 isolates (EC50 0.9–13.2 µg/mL) Inhibited infection with SARS-CoV-2 in K18-hACE2 transgenic mice (in vitro, in vivo)	[42]
Bone regeneration				
Sclerostin	High- sulfated HA (sHA3)	Non-diabetic + diabetic ZDF rats 3 mm femoral defect Lactide-based scaffolds coated with collagen/sHA3 or collagen/HA	Improved bone defect regeneration for scaffolds with collagen/sHA3 and collagen/HA in diabetic rats No improvement for non-diabetic animals Increased sclerostin binding to collagen/sHA3 coatings in vivo for diabetic rats	[83]
FGF-2	Dextran sulfate OTR4131, OTR4120	Rat mesenchymal stem cells (rMSCs)	Enhanced rMSC migration in Boyden chamber model OTR4120 but not OTR4131 increased expression of osteogenic marker genes compared to control cells in basal medium (in vitro)	[84]
Chronic wounds				
TIMP-3	sHA3, over- sulfated CS	SPR, enzyme kinetics, hydrogen/deuterium exchange mass spectrometry Human bone marrow stromal cells	Higher binding capacity of sHA3 and over-sulfated CS for TIMP-3 compared to heparin No interference of GAG-binding to TIMP-3 with MMP-1/-2 inhibition by TIMP-3 Reduced binding of TIMP-3/GAG complexes to endocytic receptor LRP-1 Accumulation of TIMP-3 in pericellular space of human bone marrow stromal cells after sHA treatment (in vitro)	[38,85]
GAG-binding growth factors (not specified)	Cacipliq20 (RGTA)	16 African American patients 18 analyzed wounds due to diabetes, pressure, vascular or burn wounds	22% full wound closure after four weeks of treatment Reduced wound sizes by 15–18% Decreased wound-related pain (60–70%) Clinical trial (prospective pilot study, each patient served as own control)	[86]
Cartilage tissue engineering				
TGF-β3, lysozyme	Fully sulfated sodium cellulose sulfate	Human mesenchymal stem cells (hMSCs)	Higher binding of TGF-β3 to gelatin scaffolds containing cellulose sulfate compared gelatin control scaffold Increased gene expression of collagen type II and staining of sulfated GAGs after hMSC pellet culture with 0.01% soluble cellulose sulfate Higher concentrations (>0.1%) of cellulose sulfate in gelatin gels decreased collagen type 2 production and reduced expression of chondrogenic marker genes (in vitro)	[87]
Neural tissue engineering				
Nerve growth factor (NGF)	Fully and partially sulfated cellulose sulfate	Dorsal root ganglion (DRG) neurons	Higher binding of NGF to gelatin-based scaffolds containing cellulose sulfate compared to those with CS Enhanced neurite extension (in vitro)	[88]

**Table 2:** Studies investigating biomedical applications of non-saccharide-based GAG mimetics.

Target Protein	GAG Mimetic	Cells/Model	Outcome/Limitation	Ref.
Inflammation				
E-selectin	GMI-1070 (rivipansel)	Male sickle cell mice SCD patients with vaso-occlusive crises	IC50 for E-selectin binding in ELISA: 4.3 µM Sustained blood flow rates and improved survival of SCD mice after surgical trauma Phase II study: compound safe in acute vaso-occlusion, reduced time to resolution of vaso-occlusive crises, significantly decreased opioid use Phase III clinical study: primary end point of readiness for discharge was not achieved; however, E-selectin was reduced 61% in the intervention group with rivipansel compared to baseline	[89,90,91]
Cancer				
Heparinase	Cyclo- phellitol- derived heparinase inhibitors	Cancer cell lines (U87 cells, B16 melanoma cells, 4T1 breast cancer cells) Murine metastasis models (in vivo)	Reduced heparinase activity Decreased cancer cell aggressiveness (proliferation/invasion) in vitro Significantly reduced metastasis in mice ECM-heparan sulfate preserved; less release of mitogens (in vitro, in vivo)	[92]
Infectious diseases				
Herpes simplex virus (HSV)	Benzene sulfonate- functionalized mesoporous silica nanoparticles	HSV-1 and HSV-2	Benzene sulfonate-functionalized mesoporous silica nanoparticles loaded with acyclovir decreased infection in plaque reduction assay Benzene sulfonate functionalization reduced acyclovir drug release (in vitro)	[93]
Bone regeneration				
GAG-binding growth factors (especially BMP-2)	Peptide amphiphile molecule (Lauryl- VVAGEGD (Kp- sulfo- benzoate)S) -Am)	Rat mesenchymal stem cells (rMSCs) Rabbit tibial bone defect model	Increased expression of osteogenic marker genes and alkaline phosphatase activity after rMSC culture on GAG mimetic peptide nanofibers Improved formation of cortical bone compared to treatment with physiological saline In vitro, in vivo, no standard of care included in bone defect model, no binding data for BMP-2 shown	[94]

## Data Availability

No new data are included.
